# Is Ethiopian community-based health insurance affordable? Willingness to pay analysis among households in South Central, Ethiopia

**DOI:** 10.1371/journal.pone.0276856

**Published:** 2022-10-27

**Authors:** Abdene Weya Kaso, Abdane Haji, Habtamu Endashaw Hareru, Alemayehu Hailu

**Affiliations:** 1 Department of Public Health, College of Health Science, Arsi University, Asella, Ethiopia; 2 Oromia Regional Health Bureau, Lemu and Bilbilo District Health Office, Bokoji, Ethiopia; 3 School of Public Health, College of Medicine and Health Science, Dilla University, Dilla, Ethiopia; 4 Bergen Centre for Ethics and Priority Setting, Department of Global Public Health and Primary Care, University of Bergen, Bergen, Norway; 5 Harvard T.H. Chan School of Public Health, Harvard University, Boston, MA, United States of America; University of Bologna, ITALY

## Abstract

**Background:**

Community-based Health Insurance (CBHI) is a voluntary prepayment mechanism that guarantees the provision of basic healthcare services without financial barriers to underserved segments of the population in developing countries. The Government of Ethiopia launched the CBHI program to protect the community from high out-of-pocket health expenditure and improve health service utilization a decade ago. However, to improve the quality of healthcare services delivery in health facilities and cover the changing costs of healthcare, the government should revise the contribution of the CBHI scheme. Therefore, we determined the willingness to pay for a CBHI scheme and associated factors among rural households of Lemu and Bilbilo district, South Central Ethiopia.

**Methods:**

We conducted a community-based cross-sectional study design to assess willingness to pay for the CBHI scheme and its associated factors among households in Lemu and Bilbilo districts, South Central Ethiopia. We used a double bounded contingent valuation method to elicit households’ willingness to pay for the CBHI scheme. Data were coded, cleaned, entered into Statistical Package for Social Science (SPSS) version 25, and exported to STATA 16 for analysis. A logistic regression analysis was conducted to determine the presence of statistically significant associations between the willingness to pay for the CBHI scheme and independent variables at a p-value <0.05 and Adjusted odds ratio (AOR) values with 95% CI. Finally, we checked the fitness of the model using Hosmer and Lemeshow’s goodness-of-fit test.

**Results:**

Of the 476 study participants, 82.9% (95% CI: 79.2%, 86.01%) were willing to pay for the CBHI scheme and only 62% of them can afford the average amount of 358.32ETB ($7.68) per household per annum. Primary education (AOR = 3.17; 95% CI: 1.74–5.80), secondary and above education (AOR = 4.13; 95% CI: 1.86–9.18), large family size (AOR = 2.75; 95% CI: 1.26–5.97), monthly income of 500-1000ETB (AOR = 3.75; 95% CI: 1.97–7.13) and distance to public health facilities (AOR = 2.14, 95% CI: 1.04–4.39 were significantly associated with willingness to pay for the CBHI scheme.

**Conclusion:**

In this study, around 83% of respondents were willing to pay for the CBHI and meet the government expectation for 2020. The study also revealed that educational status, family size, monthly income, and distance from the health facilities were significant factors associated with WTP for the CBHI scheme. In addition, we found that a large number of the respondents couldn’t afford the average amount of money that the participants were willing to pay for the CBHI scheme. So, the government should consider the economic status of the communities while revising the CBHI scheme premium not to miss those who cannot afford the contribution.

## Introduction

Financial protection during obtaining health care services is an important dimension of universal health coverage (UHC) [1–3]. However, the proportion of catastrophic health expenditure increased by 5.3% at a 25% threshold until 2015, from 100 million people to about 200 million people every year [2]. Despite the availability of prepayment mechanisms, out-of-pocket payment (OOP) remained the major means of financing the health system in low and middle-income countries (LMIC) [4]. In sub-Saharan African countries, OOP accounted for 40% of total health expenditures and exposed a significant number of communities to financial burdens that limit access to healthcare services at the point of service delivery [5]. Besides, high OOP, poor quality of healthcare services at public facilities, and low coverage of prepayment means were the major challenges of the health system of Ethiopia for a decade [6, 7]. In Ethiopia, OOP expenditures constitute 34% of total health expenditures and exposed a huge financial hardship to communities for a decade [8]. But the WHO recommends moving away from direct OOP to prepayment means and pool risks through health care financing strategies to mobilize resources for health [1]. In addition, there is an increased advocacy for the CBHI schemes as a viable option for catastrophic health expenditure in LMIC for a decade [9]. Currently, CBHI is a widely known prepayment mechanism in LMIC including Ethiopia. It helps communities to manage their healthcare costs and provides access to equitable healthcare services for the poor and other vulnerable groups [4, 10]. The government of Ethiopia has launched the CBHI program a decade ago with a nominal contribution from households, and it is highly subsidized by the government. The introduction of the CBHI scheme in the health financing reforms of 2005 generated a remarkable change in the public health facilities [11, 12].

After the reform, the introduction of the CBHI scheme has reduced catastrophic OOP expenditure, increased health care utilization, availability of drugs, and quality of services through retaining mobilized resources at health facilities [12–15]. For instance, in North-west Ethiopia, around 50.5% of insured households utilized healthcare services from public health facilities when compared to non-insured individuals (29.3%) [16]. Besides, according to the Federal Ministry of Health report during the evaluation of the CBHI scheme in piloted districts, 72.3% of the CBHI scheme members have visited health facilities [11]. Moreover, studies in Ethiopia revealed that the implementation of the CBHI scheme has reduced catastrophic health expenditure by 23.2%, increased the annual outpatient visits by 111%, and annual revenues of the facilities by 47% [10, 17, 18]. Even though the government of Ethiopia proposed to enroll more than 80% of households in the CBHI scheme by 2020, there is still a low enrollment rate in the program [11]. Currently, the households uptake of the CBHI scheme was 73%, in West Arsi zone [19], 78%, in Bench Maji zone [20], and 12.8% in Sidama Zone [21]. In addition, previous studies found that 74.8% of residents in Gemmachis district [22], 83.9% of households in Bugna district [23], and 90% of households in Jimma Zone [24] were willing to pay for the CBHI scheme. The previous findings reported that households’ willingness to pay (WTP), renew their CBHI membership, and enroll in the CBHI program depends on the affordability of premium, quality of care, waiting time to get services, and availability of basic logistics and supplies in health facilities [21, 25–28]. But to improve the quality of healthcare services in health facilities and cover the changing costs of healthcare, the government should revise the contribution of the CBHI scheme. Currently, as the previous CBHI scheme contribution and 25% government subsidies for all members were inadequate to cover the costs of healthcare services, the government of Ethiopia has revised the scheme premium from 240 Ethiopian Birr (ETB) to 410 ETB per household per annum [13]. Additionally, households’ WTP for the projected CBHI scheme played a crucial role in achieving the UHC and delivering high-quality healthcare services despite budget cuts and institutional challenges [29]. Therefore, we determined the willingness to pay for the CBHI scheme and associated factors among rural households of Lemu and Bilbilo district, South Central Ethiopia.

## Method and material

### Study setting, design, and period

This community-based cross-sectional study was conducted in Lemu and Bilbilo districts, South Central Ethiopia from September 10–30, 2021. Lemu and Bilbilo districts have 32 kebele (the smallest administrative unit) with a total population of 180,695 (89,352 men and 91,343 women) according to a 2007 Ethiopian center statistical agency report. Lemu and Bilbilo district is located 175 km from Addis Ababa, the capital city of Ethiopia, and 56 km from Asella, the zonal town of the Arsi zone. It is bordered on the South by the Hasasa district, on the West by Munesa District, on the North by Digelu and Tijo district, and on the East by the Shirka district. Besides, the district has 7 health centers and one primary hospital, which serves around 250,000 communities in nearby districts.

### Study population and eligibility criteria

All rural households in Lemu and Bilbilo districts who were not a member of the CBHI scheme were the source population, whereas all households in the randomly selected kebele were the study population. The heads of households (i.e. Mother/Father) that have stayed for more than six months in the selected kebele and who were aged 18 and above years old were included in the study. We considered heads of the households primarily for interviews from the selected households and interviewed the senior adults aged 18 and above where the households’ headswere not available. Respondents who were government workers or who were unable to respond due to their health conditionswere excluded from the study.

### Sample size determination and sampling technique procedure

**Fig 1:**
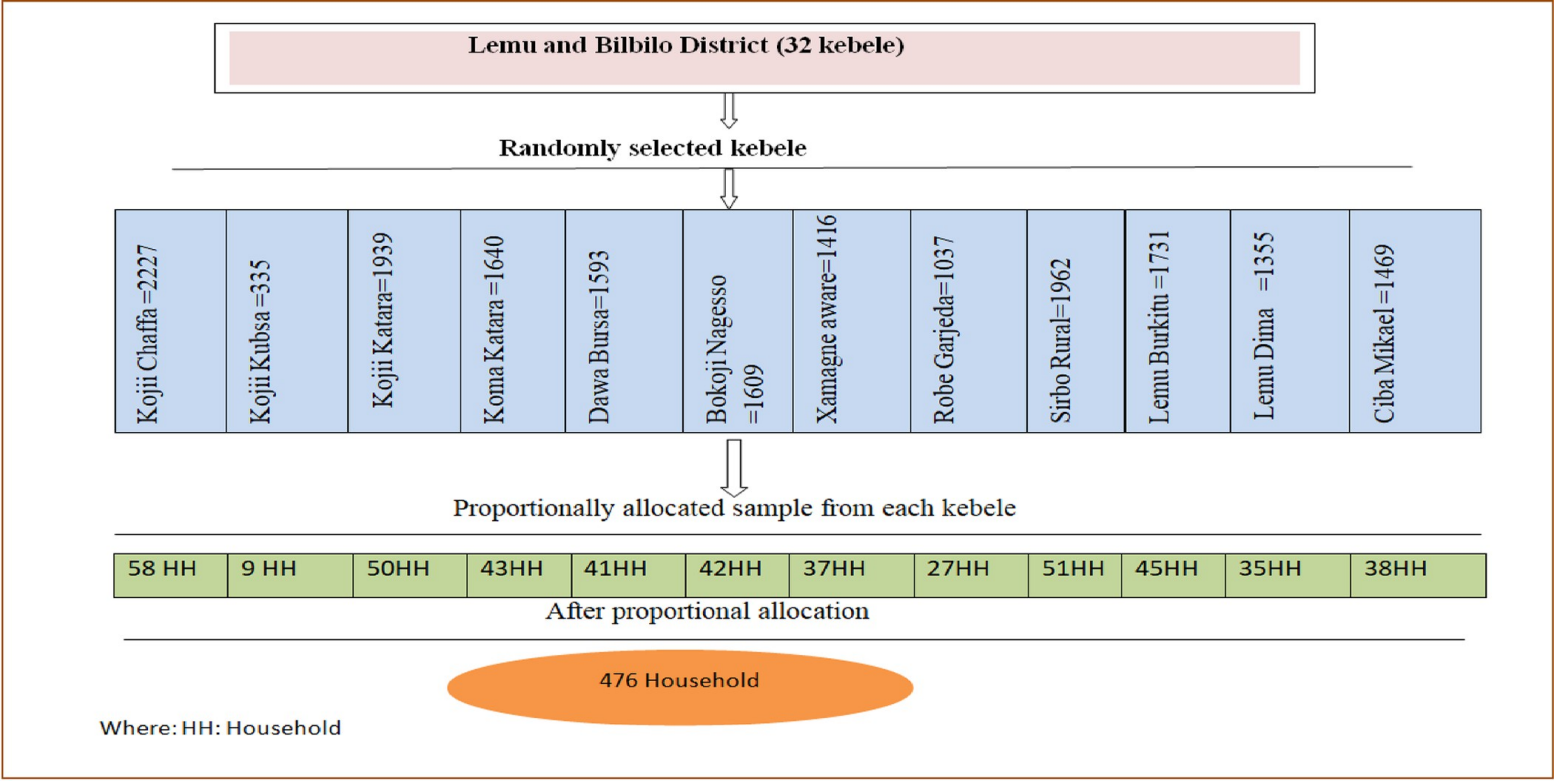
Number of sample sizes from selected kebele in Lemu and Bilbilo district, South-Central, Ethiopia, 2021.

We calculated a sample size of 476 using a single population proportion formula, taking the proportion of households WTP for the CBHI scheme (P = 74.8%) [22], 95% level of confidence, 5% marginal error, considering a design effect of 1.5 and an anticipated non-response rate of 10%. We used a two-stage sampling technique to recruit the participating households. In the first stage, we selected Lemu and Bilbilo districts from the Arsi zone as the district had a high number of households that didn’t uptake the CBHI scheme and planned to improve the CBHI scheme enrollment rate. In the second stage, we randomly selected twelve kebele out of thirty-two kebele and allocated proportionally to each kebele using the number of households residing in that administrative unit (Fig 1). A systematic random sampling method was employed to recruit the participants after we prepared a sampling frame based on the current enrollment status of the CBHI scheme.

### Variables of the study

Willingness to pay for the CBHI scheme among households in the Lemu and Bilbilo districts was the dependent variable, while factors such as socio-demographic (age, sex, family size, and marital status), socioeconomic variables (monthly income, occupation, and education status), health and health-care utilization (illness experience within the past three months, chronic illness and distance to health facilities) and awareness about the CBHI scheme were independent variables.

### Operational definition

#### Willingness to pay for the CBHI scheme

Was defined as the maximum (non-zero) amount that households heads are willing to pay for the community-based health insurance scheme, assessed using a double bounded contingent valuation method through applying a bidding game.

#### Benefits package

Was defined as the type of healthcare services provided or covered by the CBHI scheme for all eligible members of the households that had enrolled in the CBHI scheme.

#### Route of access to health facilities

Was also defined as the process through which the member of the CBHI scheme should pass to get services from high-level health facilities if other facilities existed nearby to their residential area.

### Data collection procedures and quality management

We used a pre-tested, semi-structured, interviewer-administered questionnaire to collect data from participants from September 10^th^ to 30^th^, 2021. The questionnaire was adapted from previous studies [22, 24], translated into Afan Oromo, and retranslated back into English by language experts. The questionnaire comprises socio-demographic and economic characteristics, health and healthcare utilization-related factors, awareness and perception about the CBHI scheme, and WTP for the CBHI scheme. We collected data using four trained health professionals experts who were fluent speakers of the local language. We described the hypothetical CBHI scheme in detail to the respondent including the benefits package, the criteria for membership, the potential benefits, and the terms of conditions before assessing their WTP. Thereafter the respondents were asked for their WTP for the proposed CBHI using the dichotomous choice Contingent Valuation Method (CVM). Those participants who responded “no” to the dichotomous CMV were asked for the reasons why they were refused to pay for the CBHI scheme. However, if the respondent’s response was “yes” to the dichotomous choice CVM, the bidding game was employed to ascertain the premium each respondent would willing to pay for the hypothetical scheme. In the bidding game, the respondents were asked if they would pay an initial bid amount (410 ETB) and probed the questions depending on their response to this bid. If the study participants agreed to pay the initial bid (410ETB), the interviewer would raise the bid amount to 450ETB and again question their WTP. Next, if the respondents agreed to pay for the first higher bid, the interviewer would raise the bid amount to 500ETB andagain question their WTP. Finally, if the respondents still agreed to pay for the bid, they were asked to indicate the maximum amount that they would be willing to pay using open CVM. However, if the respondent expressed unwillingness to pay the initial bid, the interviewer would lower the bid amount (350 ETB) and again question their WTP. In case the study participants disagreed to pay the first lowered bid, the interviewer would lower the bid (300ETB) andrepeat the inquiry. Lastly, if the participants still expressed unwillingness to pay for the second lowered bid, they were asked to state the maximum amount that they were willing to pay using open CVM. The principal investigator monitored and supervised the overall data collection activities and checked for completeness and consistency of data daily.

### Data analysis

The collected data were entered into SPSS version 25 and exported to STATA 16 for analysis. We utilized descriptive statistics such as frequencies and percentages for categorical variables whereas mean with standard deviation (SD) was used for summarizing continuous variables. All variables with a p-value ≤0.25 in the bivariate logistic regression analysis were fitted to a multivariate logistic regression model to determine their association with the WTP for the CBHI scheme. Odds ratios with a 95% confidence interval (CI) and a P-value less than 0.05 were reported to determine the associations between independent variablesand WTP for the CBHI scheme. Finally, the fitness of the model was assessed by using Hosmer and Lemeshow’s goodness-of-fit test.

### Ethical consideration

We obtained Ethical clearance from the Institutional Review Board of Paradise Valley University College (Ref.no.pvuc/1021/2021). We also obtained informed written consent from respondents before interviewing by explaining the purpose of the study. We assured the confidentiality of the participants’ information using a coding system and by removing any potential personal identifiers.

## Result

### Socio-demographic characteristics of the respondents

Out of the 476 study participants, 473 were included in the study with a response rate of 99.4%. Among 473 respondents, 384 (81.2%) were males and 269 (56.9%) of them were in the age category of 30–44 years old. A majority (39.5%) of the respondents attended primary education, 411 (86.9%) were married, 441 (93.2%) were Oromo by ethnicity, and 328 (69.3%) were Orthodox religious followers. More than three-fifths (67.4%) of the respondents had a family size of less than or equal to five (Table 1).

**Table 1:** Sociodemographic characteristics of respondents in South Central, Ethiopia, 2021.

Variables	Categories	Frequency (%)
Sex	Male	384 (81.2)
	Female	89 (18.8)
Age	18–29 years	127 (26.8)
	30–44 years	269 (56.9)
	Above 44 years	77 (16.3)
Marital status	Married	411 (86.9)
	Others ^a^	62 (13.1)
Religion	Muslim	115 (24.3%)
	Orthodox	328 (69.3%)
	Other ^b^	30 (6.4%)
Education	Non-formal	195 (41.2)
	Primary	187 (39.5)
	Secondary	91 (19.2)
Ethnicity	Oromo	441 (93.2)
	Other ^c^	32 (6.8)
Occupation	farmer	322 (68.1)
	Merchants	66 (14)
	Others ^d^	85 (18)
Monthly income	<500 birr	115 (24.3)
	500–1000 birr	212 (44.8)
	above 1000 birr	146 (30.9)
Family size	≤ 5	319 (67.4)
	>5	154 (32.6)

Note

^a^ = widowed/divorced

^b^ = Protestant, Catholic, Wakefta

^c^ = Amhara, Gurage

^d^ = daily labor, housewife

### Accesses to care and health-seeking behavior of respondents

Out of 473 study participants, 49 (10.4%) had chronic illnesses and 111 (23.5%) of them had experienced illnesses in the last three months. Among those who encountered illnesses in the last three months, 103 (92.8%) sought medical treatment whereas 8 (7.2%) of them didn’t seek treatment due to the difficulty of covering the cost of medical treatment. A majority (87.7%) of the respondents reside at a distance of below 11km from nearby public health facilities (Table 2).

**Table 2:** Healthcare characteristics of respondents in Lemu and Bilbilo Woreda, 2021.

Variables	Categories	Frequency (%)
Chronic illness	Yes	49 (10.4)
	No	424 (89.6)
Experienced illness in the last three months	Yes	111 (23.5)
	No	362 (76.5)
Seek treatment	Yes	103 (92.8)
	No	8 (7.2)
Place of treatment	Private clinic	20 (19.4%)
	Government health center	49 (47.6%)
	Government hospital	34 (33%)
Distance from public health facility	≤ 10km	415 (87.7)
	>10km	58 (12.3)

### Resident’s knowledge about community based health insurance scheme

Regarding respondents’ awareness of the CBHI scheme, 369 (78%) of them heard about the CBHI scheme whereas 237 (64.2%), 199 (53.9%), and 273 (74%) of the participants knew about the benefit packages, route of access and principle of the CBHI scheme. Of those who heard about the CBHI scheme, 200 (54.2%) of them reported health workers as a source of information while 72 (19.5%) of the respondents got information from family members and friends (Table 3).

**Table 3:** Respondents’ knowledge of a community-based health insurance scheme.

Variables	Categories	Frequency (%)
Heard about the CBHI scheme	No	104 (22%)
	Yes	369 (78%)
Source of information	Health workers	200 (54.2%)
	Radio and TV	73 (19.8%)
	Family and friends	72 (19.5%)
	Other	24 (6.5%)
Know benefit package	Yes	237 (64.2%)
	No	132 (35.8%)
Know Principles of CBHI	Yes	273 (74.0%)
	No	96 (26.0%)
Know the route of access	Yes	199 (53.9%)
	No	170 (46.1%)

### Respondent’s perception of community-based health insurance scheme

**Fig 2:**
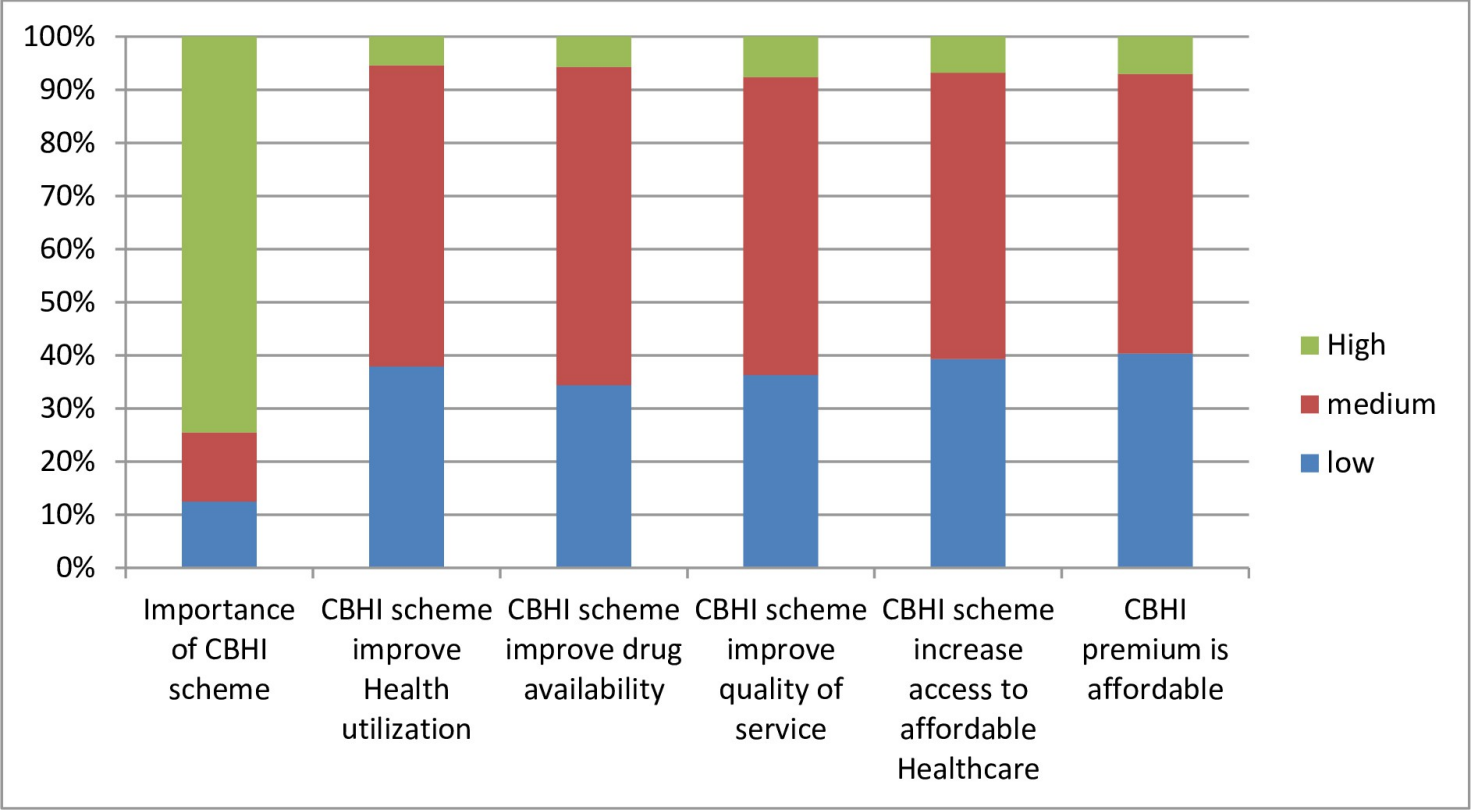
Respondents’ perception towards community based health insurance scheme, 2021.

Around 275 (74.5%) respondents think that the CBHI scheme has high importance whereas 194 (52.6%) of them perceived that the potential of affordability of the current CBHI premium was medium. The perceived potential of the CBHI scheme to increase access to affordable healthcare was medium among 199 (53.9%) of participants. Moreover, around 56.6% and 59.9% of respondents perceived the potential of the CBHI scheme to improve the healthcare utilization and drug availability as a medium respectively. Besides, the perception of households towards the potential of the CBHI scheme to improve the quality of health services was medium in 56.1% of the participants (Fig 2).

### Willingness to pay for the community based health insurance scheme

**Fig 3:**
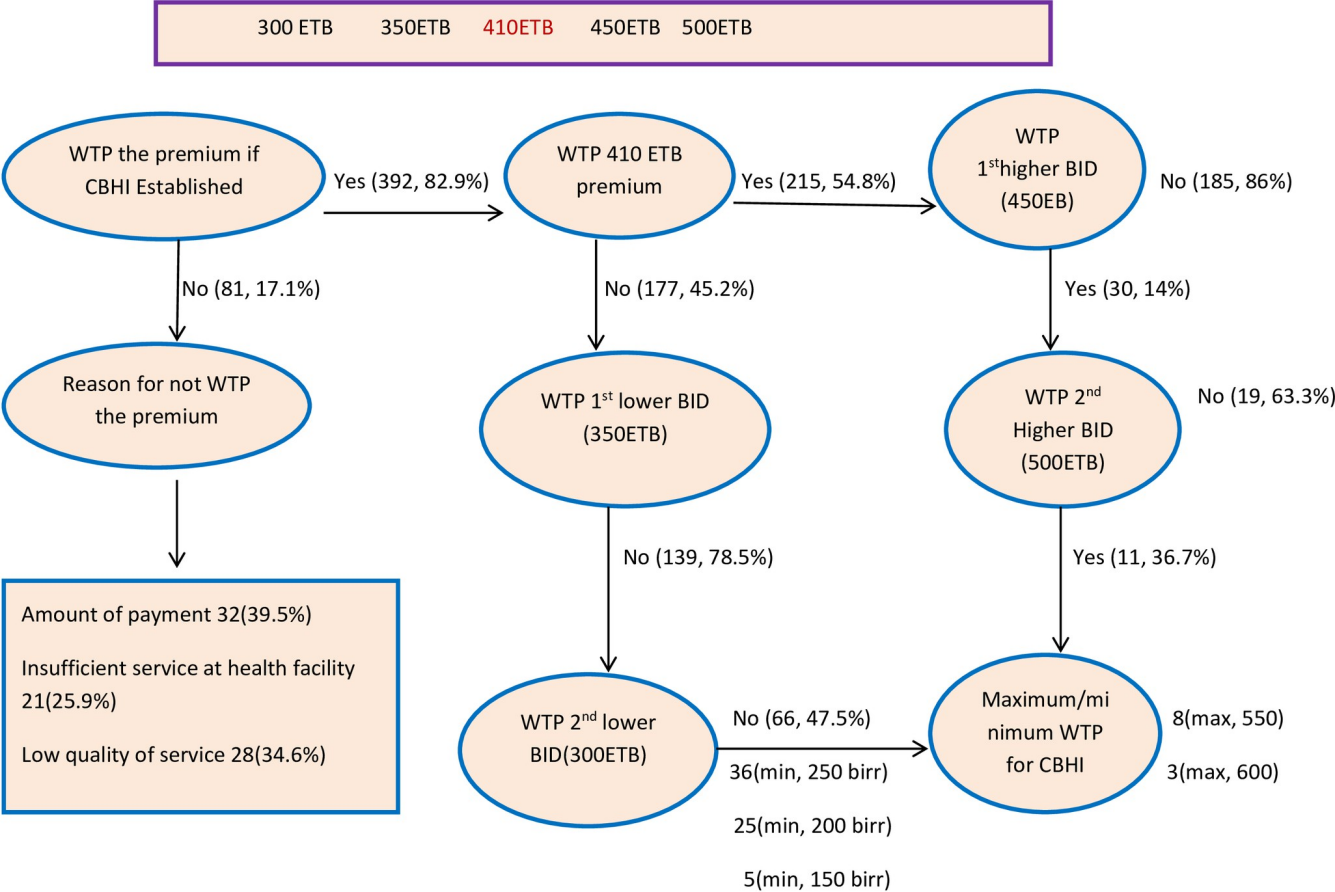
Participants’ willingness to pay for community based health insurance scheme, 2021.

Of the 473 study participants, 82.9% (95% CI: 79%, 85.9%) of them were willing to pay for the proposed community-based health insurance scheme. The mean (SD) amount of contribution the respondents were willing to pay per household per annum was 358.32 (82.13) ETB or (7.68USD) [30]. Out of 392 study participants, 215 (54.8%) of them were willing to pay the national stated premium of 410 ETB (8.78USD). Additionally, of the respondents who were willing to pay the initial bid, 30 (14%) of them were also prepared to pay the first higher bid of 450ETB (9.64USD), and 11 (36.7%) of those who were willing to pay the first higher bid were also willing to pay the 500ETB (10.71USD) bidding amount. Besides, out of the participants who were not willing to pay the initial bid, 139 (78.5%) of them were not willing to pay the lower bid of 350ETB (7.49USD) whereas 66 (47.5%) of those who were not willing to pay the first lower bid were also not willing to pay the second lower bid of 300ETB (6.42USD) bidding amount. Out of 81 study participants who were not willing to pay for the CBHI scheme; 32 (39.5%), 21 (25.9%), and 28 (34.6%) of them were not willing to pay due to amount of payment, insufficient services, and poor perceived quality of healthcare at public health facilities respectively (Fig 3).

### Respondents’ ability to pay for the annual CBHI premium

To calculate the respondent’s ability to pay the WTP amount of 358.32ETB, the health expenditure-income ratio technique was employed. Based on data from developing countries, we used a 5% health expenditure-to-income ratio, which suggests that a household that spends less than 5% of their income does not push into poverty because of healthcare costs [31–33]. Using this approach, we found that only 62% of the study participant could afford to pay for the CBHI scheme premium (Table 4).

**Table 4:** Respondents’ ability to pay for the annual CBHI premium using expenditure to the income approach.

	<5% of household income	≥5% of household income
Frequency	243	149
Percentage	62.0%	38.0%

### Factors associated with respondents’ willingness to pay for CBHI scheme

We found age, marital status, sex, educational status, occupation, family size, monthly income, experiences of an illness in the last three months and distance to the health facilities were eligible for multivariate logistic regression analysis (p-value of <0.25). In multivariate logistic regression, family size, monthly income, distance from public health facilities, and educational status of the respondents remained to have an association with WTP for the CBHI scheme. The odds of willingness to pay for the CBHI scheme among families who had a family member of greater than five was almost 3 times higher than a family member of five or less (AOR = 2.75; 95% CI: 1.26–5.97). In addition, those respondents who attended primary and above education were almost 3 times more likely to pay for the CBHI scheme than those who had informal education (AOR = 3.17; 95% CI: 1.74–5.80). Besides, the odds of willingness to pay for the CBHI scheme among individuals who had secondary and above education were 4.13 times higher than those with no formal education (AOR = 4.13; 95% CI: 1.86–9.18). Participants who had a monthly income of 500-1000ETB were almost four times more likely to pay for the CBHI scheme compared to those who had a monthly income of less than 500 ETB (AOR = 3.75; 95% CI: 1.97–7.13). Similarly, the odds of willingness to pay for the CBHI scheme among respondents who live at a distance of less than 11km from public health facilities were almost 2 times higher than those who live at a distance of 11km and above from health facilities (AOR = 2.14, 95% CI: 1.04–4.39) (Table 5).

**Table 5:** Factors associated with respondents’ Willingness to pay for CBHI scheme, 2021.

Categories	Willingness to pay		COR(95%CI)	AOR(95%CI)
	Yes (%)	No (%)		
**Age categories**				
18–29 years	101 (79.5)	26 (20.5)	1	1
30–44 years	225 (83.6)	44 (16.4)	1.35 (.79, 2.29)	1.42 (.75, 2.70)
Above 44 years	66 (85.7)	11 (14.3)	1.62 (0.75, 3.49)	0.95 (0.31, 2.95)
**Marital status**				
Married	338 (82.2)	73 (17.8)	1	1
Other	54 (87.1)	8 (12.9)	1.51 (0.69,3.30)	1.29 (0.52,3.18)
**Sex**				
Male	315 (82)	69 (18)	0.69 (0.36,1.33)	0.77 (0.33,1.83)
female	77 (86.5)	12 (13.5)	1	1
**educational status**				
Non-formal	149 (76.4)	46 (23.6)	1	1
Primary	163 (87.2)	24 (12.8)	2.06 (1.21, 3.51)	3.17 (1.74, 5.80)*
Secondary and above	80 (87.9)	11 (12.1)	2.31 (1.14, 4.70)	4.13 (1.86, 9.18)*
**Occupational status**				
Farmer	268 (83.2)	54 (16.8)	1	1
merchant	53 (80.3)	13 (19.7)	0.86 (0.44,1.68)	1.06 (0.46,2.45)
Other	71 (83.5)	14 (16.5)	1.07 (0.56,2.03)	1.25 (0.52,3.05)
**Monthly income**				
Less than 500 birr	84 (73)	31 (27)	1	1
500–1000 birr	190 (89.6)	22 (10.4)	3.48 (1.91,6.32)	3.75 (1.97,7.13)*
Above 1000 birr	118 (80.8)	28 (19.2)	1.7 (0.95,3.02)	1.66 (0.81,3.41)
**Distance to Health facility**				
≤10km	349 (84.1)	66 (15.9)	1.78 (0.94,3.39)	2.14 (1.04,4.39)*
>10km	43 (74.1)	15 (25.9)	1	1
**Family size**				
≤ 5	256 (20.2)	63 (19.8)	1	1
> 5	136 (88.3)	18 (11.7)	1.94 (1.10,3.39)	2.75 (1.26,5.97)*
**Illness in last 3 months**				
Yes	91 (82)	20 (18)	1.22 (0.71,2.10)	0.75 (0.42,1.34)
No	301 (83.1)	61 (16.9)	1	1

Note

* p-value < 0.05

## Discussion

The Ethiopian health care system is under tremendous reform to protect its citizens from catastrophic out-of-pocket health expenditure by introducing voluntary health insurance [12]. The overall aim of this study was to determine WTP for the CBHI scheme and associated factors among households in the Lemu and Bilbilo districts of Ethiopia. In this study, we found that 82.9% of the participants were willing to pay for the CBHI scheme. This finding is almost comparable with the study done in Nigeria,82% [34], Kewiot and EfratanaGedem districts, 79% [35], and Oromia Region,83.9% [36]. However, it is less than studies done in Bangladesh, 86.7% [37], Jimma Zone, 90% [24], and higher than studies in Pakistan, 64% [38], Nepal, 71.3% [39], Nigeria,74.52% [40], Bugna district,77.8% [23] and Gemmachis district, 74.8% [22]. This variation might be due to differences in the sample size, study period, socioeconomic status, and level of awareness of the participants towards the CBHI scheme. The affordability of the CBHI scheme premium had a significant influence on households’ enrolment and retention in the scheme [27, 31, 41–43]. In this study, we found that only 62% of respondents have ability to pay (ATP) the average amount of money that the participants were willing to pay for the CBHI scheme. The implication of low level of ATP of respondents despite high WTP is that there is uncertainty in improving the coverage of the schemes and attaining the goal of UHC unless there is external financial support. Therefore, the government should consider involvement of domestic donors and small microfinance in subsidising the CBHI scheme premiums as observed in other African countries [44–46]. Besides, the government should improve domestic resource mobilization to subsidize the premium so as to allow the some segment of communities to enroll in the scheme without facing further financial hardship. In addition, the mean amounts of money participants were willing to pay was 358.32ETB ($7.68) per household per annum. This is consistent with the currently proposed payment of 8.53USD [47], a study in Gemmachis district, $7.76 [22], and Jimma Zone, $8.27 [24]. However, it is higher than report from Fogera district of Ethiopia,$1.95 [48] and lower than studies from Saudi Arabia, $13.33 [49], Nigeria, $20.4 [50], Cameron, 103.44$ [51], Bugna district, $11.12 [23], Amhara Region, $10.5 [35] and Oromia Region, $17.9 [36]. The discrepancy might be due to the difference in the study period, economic growth, and socioeconomic status of study participants.

We found that study participants with large family members were more likely to pay for the CBHI scheme than those with few family members. This finding was supported by the study conducted in Nigeria [34, 52], Saudi Arabia [49], Bugna district, Ethiopia [23], Amhara Region, Ethiopia [35], and Jimma zone, Ethiopia [24]. This possible explanation might be households with large family members suffer from financial risks due to unanticipated illnesses, particularly in low-income families. So, they might have chosen to pay for the CBHI scheme to avoid the risk of catastrophic OOP health expenditure during the time of illness. The probability of willingness to pay for the CBHI scheme was higher among respondents who were more educated than those who had no formal education. This finding was supported by the study conducted in Bangladesh [37], Nigeria [50], Fogera District [48], and Gemmachis district, Ethiopia [22]. This could be because those individuals with better educational levels might have a better understanding of the concepts, benefits package, and principles of the CBHI scheme, which can ease their decision to pay for the scheme. In addition, educated respondents have a better awareness of the benefit of making regular insurance payments to avoid the risk of catastrophic healthcare expenditures at the time of unpredictable illness.

We found that respondents’ monthly income had a significant association with WTP for the CBHI scheme. Those individuals who had high monthly income were more likely to pay for the CBHI scheme than those who had low monthly income (less than 500ETB). This finding is similar with studies done in Malaysia [53], India [54], Pakistan [38], Bangladesh [37], Cameron [51], and Sierra Leone [55]. This could be because respondents with high income might have enough resources to pay for the requested CBHI scheme premium to protect their family members from unpredictable illnesses [24, 38]. We found that the distance respondents reside from the public facility had a significant effect on WTP for the CBHI scheme. The odds of willingness to pay for the CBHI scheme were almost 2 times higher among those who reside at a distance of less than 11kilometres from nearby health facilities than their counterparts. This finding was supported by the study conducted in Bangladesh [42], Nigeria [52], and Eastern Ethiopia [22]. The possible reason might be those respondents who were very close to the health facilities have less travel time and non-medical costs such as transportation, and food expenses and are more likely the use healthcare service. Even though our study had a high response rate, it has a few drawbacks. First, the use of dichotomous double bounded CVM to assess the WTP for the CBHI scheme might not indicate the actual contribution that the respondents can pay for the proposed CBHI scheme based on their own choice. Second, a significant number of study participants have no awareness ofthe route of access, principle, and benefits packages of the CBHI scheme and this might affects the respondents’ level of WTP for the proposed health insurance scheme. Third, since we included only the participants from rural area, the findings of this study cannot be generalized to households residing in urban areas.

## Conclusion

In this study, around 83% of respondents were willing to pay for the CBHI and meet the government expectation of 2020. The study also revealed that educational status, family size, monthly income, and distance from the health facilities were significant factors associated with WTP for the CBHI scheme. In addition, we found that a large number of the respondents couldn’t afford the average amount of money that the participants were willing to pay for the CBHI scheme. So, the government should consider the economic status of the communities while revising the CBHI scheme premium not to miss those who cannot afford the contribution.

## Supporting information

S1 Dataset (DTA)

S1 Questionnaire (DOC)

S2 Questionnaire PLOS inclusivity questionnaire. (DOCX)

## Abbreviations

AOR: Adjusted Odd Ratio
CBHI: Community Based Health Insurance
CI: Confidence Interval
COR: Crude Odd Ratio
ETB: Ethiopian Birr
IRB: Institutional Review Board
LMIC: Low and Medium-Income Countries
OR: Odd Ratio
USD: United State Dollar
WHO: World Health Organization
WTP: Willingness to Pay
