# Supplementary material for: Is Ethiopian community-based health insurance affordable? Willingness to pay analysis among households in South Central, Ethiopia
Source: PLoS One. 2022 Oct 27;17(10):e0276856. doi: 10.1371/journal.pone.0276856 (PMC9612585; doi:10.1371/journal.pone.0276856)
Supplement: S1 Questionnaire — (DOC) [file pone.0276856.s002.doc]

**DILLA UNIVERSITY**

**COLLEGE OF MEDICINE AND HEALTH SCIENCE**

**DEPARTMENT OF PUBLIC HEALTH**

**Consent form**

Consent form that certify the respondents agreement before the interview on assessment willingness to pay for community based health insurance scheme and associated factors among households of Lemu and Bilbilo district, South central Ethiopia, 2021

Name of kebele ________________________

Questionnaire identification number _________________

Introduction: my name is _______________________ I am interviewing Lemu and Bilbilo residents about Willingness to pay and associated factors for community based health insurance. You are selected to be one of the participants in the study. The study will be conducted through interviewer administered questionnaire. The information you give us is confident and will be used only for this study purpose. A code number will identify every participant and no names will be used. If a report of the result is published only summarized information of the total group will appear. The interview is voluntary: you have the right to participate, or not to participate or refuse to do so at any time during the interview. Your refusal will not have any effect on services that you or any member of your family receives. However your participation is important to fulfill the study gap. If there are things that require clarification please don’t hesitate to ask the facilitators for clarification.

Do you mind participating in this study, please?

1. Yes, I want to participate in the study. (Please go to the next page)
2. No, I don't want to participate in the study.
3. **Sociodemographic and socioeconomic** characteristics

| S/no | Question | Response | Skip to |
| --- | --- | --- | --- |
| 101 | Age of respondent (in complete years) | _____________ |  |
| 102 | Sex of respondent | 1. Male 2. Female |  |
| 103 | What is your Religion? | 1. Muslim  2. Orthodox  3. Protestant  4. Catholic  5. Other (specify)______ |  |
| 104 | What is your ethnicity? | 1.Oromo  2.Amhara  3.Other(specify)_______ |  |
| 105 | Current marital status of the respondent? | 1.Married  2.Divorced  3. Single  4. Widowed  5.Separeted |  |
| 106 | What is your main occupation? | 1. Farmer  2. Housewife  3. Merchant  4. Laborer  5. Student  6.Other(specify)____ _ |  |
| 107 | What is your highest educational level? | 1. Cannot read and write  2. Read and write only  3. Primary education(1-8)  4. Secondary education and above |  |
| 108 | What is your monthly income (in birr) | ----------------- |  |
| 109 | What is number of family member in the house? | 1. Less than or equal to 5 2. Greater than 5 |  |

1. **Questions about Healthcare related factors**

| 201 | Do you or other member of the household have chronic illness? | 1. Yes  2. No |  |
| --- | --- | --- | --- |
| 202 | Have any member of your family encountered any illness during the last 3 months? | 1. Yes  2. No | If “yes” go to 303, If ‘no’ skip to “301” |
| 203 | If yes, did you/they seek medical treatment for the last 12 month episode? | 1. Yes  2. No |  |
| 204 | If yes, from where did you get treatment? | 1. Self-treatment  2. Local drug vender  3. Private Heath Facility  4. Public health center  5. Public hospital  6. Traditional healer  7. Other (specify) ___ |  |
| 205 | How far your you live from the nearby health facilities | 1. Less than 5km 2. 6km-10km 3. Above 10km |  |

1. Questions regarding knowledge about CBHI scheme

| 301 | Do you heard about the community-based health insurance? | 1.Yes  2.No |  |
| --- | --- | --- | --- |
| 302 | If yes, from where did you get information? | 1. Health workers 2. Radio and TV 3. Family and friend 4. Others(Specify)-------- |  |
| 303 | Do you know the principle of CBHI? | 1.Yes  2.No |  |
| 304 | Do you know the benefit package of CBHI? | 1. Yes 2. No |
| 305 | Do you know the route of access to health services? | 1. Yes 2. No |  |

1. Questions about households perception about CBHI

| 306 | How did you perceive the ability of CBHI to make health more affordable? | 1. Low  2. Medium  3. High |  |
| --- | --- | --- | --- |
| 307 | How did you perceive the potential of CBHI increasing access to affordable healthcare? | 1. Low  2. Medium  3. High |  |
| 308 | How did you perceive potential to CBHI improve household utilization of the health care service? | 1. Very low  2. Medium  3. High |  |
| 309 | How did you perceive the CBHI potential to improve quality of services provided by? | 1. Low  2. Medium  3. High |  |
| 310 | How did you perceive Potential of CBHI to ensure constant availability of drugs at facilities? | 1. Low  2. Medium  3. High |  |
| 311 | How did you perceive the importance of CBHI scheme to you  ? | 1. Low  2. Medium  3. High |  |

1. **Questions about Willingness to pay for CBHI**

**Scenario**

Suppose one of your family member get ill and needs immediate medical care at health facilities. However, the cost of providing healthcare at both private and public health facilities is increasing at alarming rate. But you faced budget constraint and need to borrow from relatives or sell your assets to cover cost of the medical care. Besides, sometimes due to prolonged delay in seeking care as a result of budget constraints, the sick individual may suffer from illnesses and even get sudden death. Since the World health organization declared the universal health coverage, the government of Ethiopia decided to protect its rural households from financial problem at the time of service delivery and improve access to quality health care. However, due to the growing cost of providing healthcare, the government may not be able to provide all basic healthcare at minimum cost for all communities to attain the goal of universal health coverage, Thus, the federal Ministry of Health has decided to set up the community based health insurance (CBHI) scheme in which households are required to make regular contributions. The proposed CBHI scheme covers surgery such as caesarean section and excludes dental extraction, circumcision and plastic surgery. In addition, the scheme didn’t cover dialysis and medical treatment from abroad. But your will get services provided at outpatients and inpatient services such as consultation, inpatient stay, laboratory and diagnostic investigations, and drugs for a period of one year for your family members (the head of household, the spouse and children age of less than 18 years). If you joined the CBHI scheme, you will pay the annual premium of 410 birr (400 for membership and 10 birr for registration). Organization of the insurance agency at district level will manage the scheme and have contractual agreement with public health facilities on behalf of the scheme members. Once you enrolled in the CBHI scheme, you don’t need to pay money to see a doctor for the services that are covered by the insurance. However, you will have to pay for healthcare services that were not covered by the CBHI scheme. In addition, the premium is not refunded for those who don’t need to use health services in that year. The premiums will be kept in a bank and the insurance agency will pay for the service provider on quarter basis after auditing the services provided as per contractual agreement between the two organizations. An annual audit will ensure that funds are used rationally. The insurance agency will give a financial report of the CBHI scheme to local government every year.

| 401 | Do you want to pay for CBHI scheme for established in your area? | 1. Yes 2. No | If yes go to 402, if no go to “409 |
| --- | --- | --- | --- |
| 402 | Do you want to pay the 400ETB premium? | 1. Yes 2. No | If yes” go to 403, if no” go to 406 |
| 403 | Do you want to pay 450 premiums? | 1. Yes 2. No | If yes go to  “404” |
| 404 | Do you want pay 500 premium? | 1. Yes 2. No | If yes go to 405 |
| 405 | What is the maximum amount you are willing to pay? | ___________ETB |  |
| 406 | Do you want to pay 350ETB contribution? | 1. Yes 2. No | If “no” go to 407 |
| 407 | Do you want to pay 300 ETB contributions? | 1. Yes 2. No | If no” go to 408 |
| 408 | What is the minimum amount you are willing to pay? | ___________ETB |  |
| 409 | What is your reason for not willing to pay for the scheme? | 1. Amount of payment  2.Duration of payment  3. Insufficient services at public health facilities  4. low quality of care at public health facilities  5. Other (specify) _________ |  |

Thank you for your response!
